# Effect and Safety of Mycophenolate Mofetil or Sodium in Systemic Sclerosis-Associated Interstitial Lung Disease: A Meta-Analysis

**DOI:** 10.1155/2012/143637

**Published:** 2012-05-10

**Authors:** Argyris Tzouvelekis, Nikolaos Galanopoulos, Evangelos Bouros, George Kolios, George Zacharis, Paschalis Ntolios, Andreas Koulelidis, Anastasia Oikonomou, Demosthenes Bouros

**Affiliations:** ^1^Department of Pneumonology, University Hospital of Alexandroupolis, Democritus University of Thrace, 68100 Alexandroupolis, Greece; ^2^Department of Rheumatology, University Hospital of Alexandroupolis, Democritus University of Thrace, 68100 Alexandroupolis, Greece; ^3^Laboratory of Pharmacology, Democritus University of Thrace, 68100 Alexandroupolis, Greece; ^4^Department of Radiology, University Hospital of Alexandroupolis, Democritus University of Thrace, 68100 Alexandroupolis, Greece

## Abstract

*Background*. Interstitial lung disease (ILD) is the most common complication of systemic sclerosis (SSc) with treatment ineffective. Objective: The aim of this meta-analysis was to provide an estimate of the safety and efficacy profile of Mycophenolate Mofetil (MMF) or sodium (MMS) in SSc-ILD patients. *Materials and Methods*. All studies were reviewed systematically. The main end-points were safety and efficacy profile as estimated by forced vital capacity (FVC)% and diffusion capacity of the lung for carbon monoxide (DL_CO_)% of the predicted normal value (%pred.) before and after treatment in patients with SSc-ILD. Quality assessment and data extraction were performed independently by two reviewers. *Results*. Seventeen studies were reviewed systematically. Six studies, one prospective, were eligible for analysis encompassing 69 patients, including 10 subjects from our, yet unpublished, retrospective study. There was no statistically significant difference in both efficacy outcomes of interest, including FVC% pred. (weighted mean difference 1.48, 95% confidence interval (CI): −2.77 to 5.72, *P* = 0.49) and DL_CO_ % pred. (weighted mean difference −0.83, 95% CI: −4.75 to 3.09, *P* = 0.93). No cases of clinically significant side effects were documented. *Conclusions*. Meta-analysis data suggest that MMF is a safe therapeutic modality which was associated with functional stabilization in patients with SSc-ILD.

## 1. Introduction

Interstitial lung disease (ILD) is one the most common complications of systemic sclerosis (SSc) with a prevalence of 40%–84% and represents the major source of morbidity and mortality [1–5]. Thus, lung involvement has been the target of several clinical studies estimating safety and efficacy of a significant number of therapeutic agents, including corticosteroids and cyclophosphamide [6–11]. So far, only the latter has been proven of some benefit in patients with SSc-ILD [12], as has been demonstrated in a large multicenter-randomized controlled clinical trial. Nevertheless, follow-up studies reported a rather temporary beneficial functional effect that began to partially fade 6 months after drug discontinuation [13]. In addition, assessment of HRCT findings in the same cohort of patients recorded amelioration of the extent of fibrosis in the cyclophosphamide arm [14–16]. Despite relative enthusiasm arising from the above data, the modest and temporary functional and radiological improvement in patients under cyclophosphamide treatment coupled with the potential toxicity associated with drug usage, raises crucial dilemmas of whether and for how long all patients with SSc-ILD should be treated with aggressive cytotoxic drugs and whether we should reserve these regimens for patients at greatest risk for progression and search for safer alternatives in mild-to-moderate disease patterns.

Mycophenolate mofetil (MMF) and mycophenolate sodium (MS) are commercialised drugs containing the active moiety of mycophenolate acid, an inhibitor of lymphocytes proliferation acting through blockage of inosine monophosphate dehydrogenase and interference with purine biosynthesis, that is commonly used to prevent rejection following solid-organ transplantation as well as for the treatment of several autoimmune and renal disorders [17–21]. In addition to its anti-inflammatory activity, MA acts also as anti-proliferating agent by downregulating the expression of several fibrotic growth factors such as transforming growth factor (TGF)-*β*, evidence that makes it an attractive candidate drug for the treatment of fibrotic lung diseases of different causes [22, 23]. Currently, its utility has been investigated in the context of one prospective [24] and four retrospective studies [25–28] encompassing, in total, 59 patients with SSc-ILD of mild-to-moderate disease severity, and a modest beneficial effect in functional and radiological status has been demonstrated.

While awaiting for the results of the, only so far, large multicentric-randomized clinical trial (Scleroderma Lung Study II) to compare the functional effect of MMF with oral cyclophosphamide in patients with SSc-ILD, we performed a meta-analysis of the current knowledge coupled with results from our, yet unpublished, retrospective cohort, to provide a more rigid estimate of the safety and efficacy profile of MMF and MS in SSC-ILD patients.

## 2. Materials and Methods

### 2.1. Study Selection

A MEDLINE, Embase, Ovid, and Cochrane database search was performed on all studies between 2006 and 2011 comparing the safety and efficacy of the administration of mycophenolate mofetil or sodium in patients with systemic sclerosis-associated interstitial lung disease. The following Mesh search headings were used: mycophenolate mofetil, mycophenolate sodium, scleroderma, systemic-sclerosis, interstitial lung disease, effect, safety, and lung function. The related article function from PubMed was used to broaden the search, and all abstracts, studies, and citations scanned were reviewed. No language restrictions were made. The latest date for this search was September 1, 2011. We have also enrolled in the pooled published data results from our, yet unpublished, retrospective study of the safety and efficacy profile of a 12-month MMF treatment in SSc-ILD patients.

### 2.2. Data Extraction

Two reviewers (AT and DB) independently extracted the following from each study: first author, year of publication, study population characteristics, study design, inclusion and exclusion criteria, and male-to-female ratio.

### 2.3. Inclusion Criteria

To be included in the analysis, studies had to (1) compare functional data including FVC% and DL_CO_% of the predicted normal value prior and at least 6 months after MMF or MS treatment, (2) report at least one of the outcome measures mentioned below; (3) clearly document MMF or MS administration in patients with SSc-ILD. When two studies were reported by the same institution and/or authors, they were included only if there was no overlap between the results of the studies. Otherwise, the larger higher-quality studies were included in the analysis. 

#### 2.3.1. Exclusion criteria

Studies were excluded from the analysis if: (1) it was impossible to extrapolate or calculate the necessary data from the published results that is absent from spirometry raw data; (2) there was considerable overlap between authors, centers, or patient cohorts evaluated in the published literature.

### 2.4. Outcomes of Interest

The following outcomes were used to compare the effect of mycophenolate mofetil or sodium in the same group of patients with systemic sclerosis-associated interstitial lung disease who were firstly off treatment and then administered the drug:

1. safety profile as assessed by cases of clinically significant infection, leucopenia, or elevated liver enzymes;
2. efficacy profile as assessed by functional data including FVC% and DL_CO_% of the predicted normal value.

### 2.5. Statistical Analysis

The meta-analysis was performed in line with recommendations from the Cochrane Collaboration and the Quality of Reporting of Meta-analyses guidelines. Weighted mean difference (WMD) was used to analyze continuous variables. It was reported with 95% confidence intervals (CIs). WMDs summarize the differences between the two groups with respect to continuous variables, accounting for sample size. Statistical algorithms were used to calculate the standard deviations (SDs) for studies that presented continuous data as means and range values, thus standardizing all continuous data for analysis. Analysis was conducted by use of Review Manager version 5.0.14 (The Cochrane*∖*Collaboration, Software Update, Oxford, UK). Results were analyzed by paired Student's *t*-test.

## 3. Results

### 3.1. Eligible Studies

By using the search key words listed above, we identified 50 publications. Thirty-four studies were excluded after title and abstract review. These included 14 review articles, one study in experimental model of scleroderma, and 19 letters or case reports. The remaining 16 articles were carefully evaluated and eleven were referring to studies estimating the safety and efficacy profile of MMF either in diffuse cutaneous systemic sclerosis or in other connective tissue disorders including systemic sclerosis, rheumatoid arthritis, lupus erythematosus, and polymyositis and, therefore, were excluded from further analysis since it was impossible to extrapolate or calculate the necessary data from the published results. A total of five studies encompassing one prospective [24] and four retrospective studies [25–28] evaluating safety and efficacy of MS and MMF, retrospectively, in an overall of 59 patients with SSc-ILD were included in this meta-analysis. We have also included 10 patients with SSc-ILD from our retrospective, yet unpublished, study to estimate the safety and effect of a 12-month oral administration of MMF. All patients included in the meta-analysis met American College of Rheumatology Criteria for SSc and had evidence of SSc-ILD based on HRCT findings with no other apparent cause for ILD.

As depicted in Table 1, the majority of patients enrolled in the studies included in meta-analysis were middle-aged, women of mean age 53 years old, with a mean time of SSc diagnosis and study enrolment, meaning drug initiation, ranging from 2–7.7 years. In addition, 26/42 patients (62%) were under cytotoxic treatment with either cyclophosphamide and/or azathioprine prior MMF administration. There was no data available regarding this issue for the remaining 27 patients included in the studies of Zamora et al. [28] and Koutroumpas et al. [26].

All of the studies included presented with major limitations due to the limited number of patients enrolled and their retrospective single-center nature (apart one prospective) and, therefore, their power to identify important differences in efficacy outcomes such as functional parameters could be questioned. As it is easily understandable, none of the studies was randomized, controlled evidence that further diminished the scientific rigidity of the data extracted. Five studies, all retrospectives, estimated safety and efficacy profile of MMF, while the remaining one prospective study evaluated similar outcomes of interest in SSc-ILD patients after a 12-month oral administration of MS.

### 3.2. Safety Outcomes

In the five retrospective studies no cases of liver toxicity, clinically significant infection and leucopenia were recorded during MMF treatment. In addition, MMF was well tolerated by the vast majority patients with development of nausea that led to drug discontinuation in only one patient and abdominal pain and nausea that were transient and required no further interventions in another patient. There was only one case of a patient presented *Aspergillus terreus* pulmonary infection that required treatment with voriconazole and MS suppression. She did not require admission and recovered completely. No other adverse effects were noted. The above data suggest that MMF or MS present with a readily acceptable safety and tolerability profile (Table 1). 

### 3.3. Efficacy Outcomes

**Figure 1:**
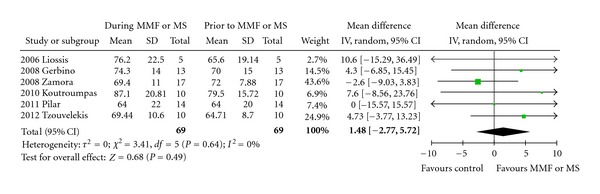
Forest plot of pooled data on FVC prior (favours control arm) and during treatment with MMF or MS (favours MMF or MS arm). Abbreviations: CI: confidence interval, DL_CO_: diffusing capacity for carbon monoxide, MMF: mycophenolate mofetil, MS: mycophenolate sodium, and SD: standard deviation.

**Figure 2:**
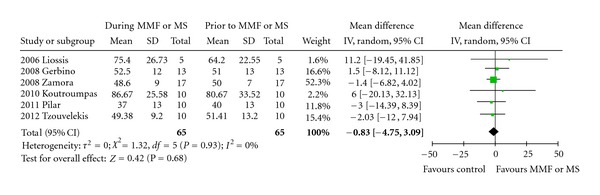
Forest plot of pooled data on DL_CO_ prior (favours control arm) and during treatment with MMF or MS (favours MMF or MS arm). Abbreviations: CI: Confidence Interval, DL_CO_: Diffusing Capacity for carbon monoxide, MMF: Mycophenolate mofetil, MS: Mycophenolate Sodium, SD: Standard deviation.

Four studies, all retrospective, (Tzouvelekis et al. mean difference of 4.73%, and 64.71% versus 69.44% of the predicted normal value from baseline, or 215 mL, CI: −7 to 1.4%, *P* = 0.001) and (Liossis et al. [27], Gerbino et al. [25] and Koutroumpas et al. [26]) reported statistically significant differences in FVC% predicted at baseline and 12 months after treatment with MMF. In the remaining two trials, a disease stabilization as assessed by nonstatistically significant differences in FVC% predicted at baseline and 12 months of treatment with MMF (and Zamora et al. [28]) or MS (Simeon-Aznar et al. [24]) (Figure 1, Table 2). With regards to DL_CO_, all studies, except of Liossis et al. [27] who reported a statistically significant improvement 6 months after MMF administration (75.4% pred. versus 64.2% pred., *P* = 0.033), clearly demonstrated nonstatistically significant alterations either increase (Gerbino et al. [25] 52.5% pred. versus 51% pred., Koutroumpas et al. [26] 86.67% pred. versus 80.67% pred., Zamora et al. [28] 51.4% pred. versus 50% pred.) or decrease (Tzouvelekis et al. 51.41% pred. versus 49.38% pred. and Simeon-Aznar et al. [24] 40% pred. versus 37% pred.) of DL_CO_ following MMF or MS oral administration compared to baseline (Figure 2, Table 2). As depicted in Table 2, all included studies enrolled patients with mild-to-moderate disease severity as assessed by functional parameters prior MMF or MS administration (FVC ranging from 64–79.5% pred. and DL_CO_ ranging from 40–64.2% pred.).

Despite the above findings, meta-analysis of the data showed no statistically significant difference favoring MMF or MS administration in both FVC% pred. (weighted mean difference 1.48, 95% confidence interval (CI): −2.77 to 5.72, *P* = 0.49) and DL_CO_% pred. (weighted mean difference −0.83, 95% CI: −4.75 to 3.09, *P* = 0.93).

## 4. Discussion

This is the first meta-analysis in the literature reporting the safety and efficacy profile of MMF and MS administration in patients with SSc-ILD. Pooled extracted data by detailed review of 6 eligible studies, encompassing 69 patients, clearly demonstrated that MMF and MS are safe therapeutic modalities, and their administration was linked with disease stabilization regarding functional parameters in patients with SSc-ILD.

Interstitial lung disease commonly complicates with various radiological, functional and histopathological patterns of disease severity, the lung of scleroderma patients and currently represents the leading cause of morbidity and mortality [1, 2, 4, 5, 29]. Its natural history is greatly downhill with therapeutic options limited and yet ineffective. So far, there is only one randomized controlled trial showing a modest beneficial effect of 2.53% in the mean absolute difference in adjusted 12-month FVC% predicted following oral cyclophosphamide therapy [12]. The above evidence of modest and temporary effectiveness of cyclophosphamide coupled with substantial drug toxicities over time highlight the need to search for safer alternatives especially for younger patients with mild disease that would benefit from longitudinal administration of therapeutic modalities with minimal side effects and reserve more aggressive and potentially more beneficial cytotoxic regimens for later stages of the disease course.

MMF and a newly commercialised delayed-release tablet containing mycophenolate acid, called MS, may represent such options. Based on the versatile anti-inflammatory, antifibrotic [22, 23] and immunomodulatory properties of its active metabolite, mycophenolic acid, MMF and MS treatments have been recently applied with promising results in patients with SSc and interstitial lung involvement [24–28, 30–32]. Although most of these studies reported a beneficial effect of MMF or MS in patients with SSc-ILD; however, all of them were unicentric, underpowered with limited number of patients enrolled, retrospective and nonrandomized controlled. Therefore, rigid conclusions regarding MMF or MS safety and efficacy profile cannot be drawn based on these studies.

While anticipating the results of the, only so far, large multicentric-randomized clinical trial (Scleroderma Lung Study II) to compare the beneficial effect in lung function parameters of a 2-year course of MMF with those of a 1-year course of oral cyclophosphamide, in patients with symptomatic scleroderma-related ILD, we performed a meta-analysis of the current knowledge coupled with results from our, yet unpublished, retrospective cohort, to provide a more rigid estimate of the safety and efficacy profile of MMF and MS in SSC-ILD patients.

After scrutinized review of the literature and using outcomes of interest safety and functional efficacy profile of mycophenolate acid in patients with scleroderma-associated ILD, a total of 6 studies fulfilling our inclusion criteria, encompassing a total number of 69 patients were enrolled in the final meta-analysis.

With regards to safety outcomes, all studies demonstrated an excellent safety profile for MMF or MS, and this finding was further supported by pooled analysis since there were only 3 patients presenting with side effects resulting in only case to drug discontinuation and in another to treatment suppression.

Regarding drug efficacy, despite the fact that 4 out of 6 studies reported on the primary analysis statistically significant functional improvement following a 12-month oral administration of MMF or MS, and the remaining two stated disease stabilization; nevertheless, pooled analysis failed to corroborate this finding. In particular, in the overall analysis of 69 patients, MMF or MS treatment failed to be associated with a statistically significant beneficial functional effect as assessed by both FVC and DL_CO_.

On the other hand, the latter observation merits further investigation since by analyzing data one can easily report that MMF or MS usage resulted in disease stabilization or reduced rate of annual functional decline compared to prior treatment with cytotoxic agents including cyclophosphamide and/or azathioprine. Meta-analysis demonstrated a mean difference of 1.48% and −0.83% of the predicted normal value in FVC and DL_CO_, respectively, while at the same time in three studies by Gerbino et al., Tzouvelekis et al., and Simeon-Aznar et al. a decline of 5.4%, 6.45%, and 6% in FVC at baseline and 12 months after administration of cytotoxic agents was reported, indicating a rather beneficial effect of MMF usage. Although a direct comparison of these percentages was impossible to be performed in this meta-analysis due to the fact that they reflect mean differences, and they do not represent raw data; nevertheless, these results may potentially indicate a favorable outcome associated with MMF administration. In line with this, given the fact that the reported annual rate of lung function deterioration over time for patients with SSc-ILD is 32% loss for VC during the first two years of the disease, 12% for 2–4 years and 3% for 4–6 years [33], it is conceivable to state that a marginal increase in FVC or even a stabilization of functional status through disease course is of vital importance for this category of patients especially when the therapeutic agent used presents with an excellent safety profile tested on a longitudinal basis.

One final issue to be clarified in our meta-analysis was the absence of a beneficial effect of MMF or MS treatment in DL_CO_ values while at the same time, as reported previously, a clear trend towards FVC improvement following mycophenolate acid administration was highly notable. At this point it is of critical importance to highlight the major problems arising when interpreting treatment effects and using DL_CO_ as an end-point for interstitial lung disease in SSc, since this variable is so often influenced by other confounding factors closely related to vascular issues including pulmonary hypertension. In particular the two largest studies [8, 11], so far, estimating efficacy of a therapeutic agent, namely cyclophosphamide, in patients with SSc-ILD, demonstrated an almost marginal statistically significant difference in FVC levels favoring drug usage, while no beneficial effect in gas-transfer indicators was reported in both studies. Our personal view is that gas-transfer capacity should not be used as primary outcome of interest in clinical trials estimating drug efficacy in patients with SSc-ILD, since major data misinterpretations may easily arise given the high incidence of pulmonary hypertension (almost 50%) that so often complicates disease natural course and influences functional parameters such as DL_CO_. Alternatively, if used, results should be cautiously concluded and extrapolated according to the presence and the severity of right-heart dysfunction.

Despite relative enthusiasm arising from the above observations, our review presents with some limitations that should be addressed cautiously. The quality of the evidence was limited; no study met all standard quality criteria since all of the studies were nonrandomized controlled, unicentric and underpowered with limited number of patients enrolled, and therefore their power to identify important differences in efficacy outcomes such as functional parameters could be questioned. Furthermore, it is important to underline that all the included studies enrolled middle-aged female patients with mild-to-moderate disease severity as assessed by functional parameters (FVC ranging from 64–79.5% pred. and DL_CO_ ranging from 40–64.2% pred.) prior MMF or MS administration. Thus, based on our data it is rather unknown whether stabilization of functional parameters could be attributed to therapeutic intervention or the former simply represents a bystander of disease clinical course. Alternatively, the majority of the patients included in the meta-analysis could be considered as slow progressors regarding their lung involvement indicating a potentially more favorable prognosis irrespective of therapeutic approach.

In conclusion, based on cumulative data from this meta-analysis our statement is that MMF or MS could wonderfully couple cyclophosphamide in the treatment of patients with SSc-ILD, since administration of the latter has been starkly demonstrated to be associated with statistically significant functional improvement in patients with SSc-ILD. On the other hand, mycophenolate acid could be administered as maintenance treatment, since it seems to represent a safe therapeutic agent that has been linked with disease stabilization regarding functional parameters overcoming the fear for potential side effects arising from longitudinal administration of aggressive cytotoxic agents or limited for patients with mild disease pattern. Slowing down disease progression and modulating disease natural history with less cost seems to be of paramount significance for this dismal disease. Larger randomized controlled studies are sorely needed to support this premise.

## Figures and Tables

**Table 1:** Baseline characteristics of the patients included per study.

Study/year	Number of patients	Age (years)	Female	Prior cytotoxic treatment received	Diffuse SSc	Disease duration (years)
Liossis et al., 2006 [27]	6	46	4/6	1/6	4/6	3.4
Gerbino et al., 2008 [25]	13	52	5/13	9/13	9/13	5
Koutroumpas et al., 2010 [26]	10	59	8/10	NA	10/10	7.7
Zamora et al., 2008 [28]	17	51	10/17	NA	15/17	2
Simeon-Aznar, 2011 [24]	14	54	13/14	10/14	8/14	6.5
Tzouvelekis et al., 2012	10	56	4/10	6/10	10/10	1.5

Total	69	53	44/69	26/42	56/69	4.7

Data are presented as mean unless otherwise stated.

Abbreviations: SSc: systemic sclerosis and NA: nonapplicable.

**Table 2:** Extracted data on outcomes of interest from all studies.

Study/year	Number of patients	Design	Side effects	FVC		DL_CO_	
				Prior MMF/MS	During MMF/MS	Prior MMF/MS	During MMF/MS
Liossis et al., 2006 [27]	6	RT	0/6	65.6 (19.14)	76.2 (22.5)	64.2 (22.55)	75.4 (26.73)
Gerbino et al., 2008 [25]	13	RT	2/13	70 (15)	74.3 (14)	51 (13)	52.5 (12)
Zamora et al., 2008 [28]	17	RT	0/17	72 (7.8)	69.4 (11)	50 (7)	48.6 (9)
Koutroumpas et al., 2010 [26]	10	RT	0/10	79.5 (15.72)	87.1 (20.81)	80.67 (33.52)	86.67 (25.58)
Simeon-Aznar et al., 2011 [24]	14	PT	1/14	64 (20)	64 (22)	40 (13)	37 (13)
Tzouvelekis et al., 2012	10	RT	0/10	64.71 (8.7)	69.44 (10.6)	51.41 (13.2)	49.38 (9.2)

Total	69		3/69				

Data are presented as mean (SD) unless otherwise stated.

Abbreviations: DL_CO_: diffusing capacity for carbon monoxide, FVC: forced vital capacity, RT: retrospective, PT: prospective, MMF: mycophenolate mofetil, and MS: mycophenolate sodium.

## List of Abbreviations

DL_CO_: Diffusion capacity of the lung for carbon monoxide
FVC: Forced vital capacity
HRCT: High-resolution computed tomography
ILD: Interstitial lung disease
MMF: Mycophenolate mofetil
MS: Mycophenolate sodium
NA: Nonapplicable
SSc: Systemic sclerosis
WMD: Weighted mean difference.
